# Femoral Head Autograft to Manage Acetabular Bone Loss Defects in THA for Crowe III Hips by DAA: Retrospective Study and Surgical Technique

**DOI:** 10.3390/jcm12030751

**Published:** 2023-01-17

**Authors:** Cesare Faldini, Matteo Brunello, Federico Pilla, Giuseppe Geraci, Niccolò Stefanini, Leonardo Tassinari, Alberto Di Martino

**Affiliations:** 1 Ist Orthopaedic Department, IRCCS—Istituto Ortopedico Rizzoli, Via Giulio Cesare Pupilli, 1, 40136 Bologna, Italy; 2 Department of Biomedical and Neuromotor Science—DIBINEM, University of Bologna, 40136 Bologna, Italy

**Keywords:** hip dysplasia, Crowe III, bone defect, autograft, femoral head, direct anterior approach (DAA), total hip arthroplasty

## Abstract

**Introduction:** The pathologic anatomy of Crowe III is characterized by the erosion of the superior rim of acetabulum, with a typical bone defect in its supero–lateral portion. The performance of a total hip arthroplasty requires the management of the acetabular bone defect, and femoral head autograft can be a valid option to optimize implant coverage. **Material and Methods:** In all, eight Crowe III patients (nine hips), seven of which having unilateral hip affected, and one with bilateral involvement by secondary osteoarthritis in DDH; maximum limb length discrepancy (LLD) of 3.5 cm in unilateral patients. All were operated on by direct anterior approach. Patients were evaluated in terms of clinical, surgical, and radiological (center-edge, horizontal coverage, cup inclination) parameters. **Results:** Cup placement was implanted with a mean of 39.5 ± 7.5°. Stem alignment showed average 1.5 ± 2.3° in valgus. LLD showed an overall average preoperative of −29.5 ± 10.5 mm at the affected side, with a significant improvement to −2.5 ± 6.4 mm (*p* = 0.023). The mean initial coverage evaluated like a percentage of the horizontal bone host was 52.1 ± 7.1%, while the mean final coverage at the last post-operative X-ray from femoral autograft bone was 97.0 ± 4.5% with an average improvement of 44.5%. Average CE improved from −9.5 ± 5.2° (CE I) to the immediate post-operative (CE II) of 40.6 ± 8.2°. At the final follow up, CE III showed a mean of 38.6 ± 6.2°, with an average decrease of 2.0°. **Discussion:** Acetabular bone defect in Crowe III DDH patients undergoing THA by DAA, can be efficiently managed by massive autograft femoral head, which allowed an adequate and long-lasting coverage of the implant, with cup positioning at the native acetabulum.

## 1. Introduction

Outcomes of developmental dysplasia of the hip in the adult patient are heterogeneous, making total hip replacement performance difficult for the altered shape of the acetabulum, which is shallow and deformed. Crowe III hips, classified by Crowe et al. in 1979 [1] as the subluxation between 75% to 100% of the femoral head on the acetabulum, represent one peculiar challenge to perform total hip arthroplasty (THA) [2]. The pathologic anatomy of Crowe III is characterized by the dislocation of femoral head that progressively erodes the superior rim of acetabulum, with a typical bone defect in its supero–lateral portion, described at the posterior border [3]. Those patients present less bone stock compared to others affected by Crowe IV deformity, where the total dislocation of the hip preserves the true acetabulum. Hip replacement in Crowe III patients can be performed in two ways, namely, using a high hip center technique, or by restoring the anatomic center of rotation (COR) at the true acetabulum [4,5,6]. The high hip center technique maintains COR in the false acetabulum; it is associated to some advantages because it reduces the risk of neurologic complications and avoids the performance of a shortening femoral osteotomy, reducing associated risks of intraoperative complications and operative time [7,8,9]. However, the high hip center technique is associated to worse biomechanics, and studies have shown that the placement of the cup at the superolateral rim could result in accelerated polyethylene wear, decreased abductor moment arm, and component loosening [10].

Restoring the anatomic hip center at true acetabulum reduces the loads, improves hip biomechanics, and supports a more physiological gait, restoring the limb length in the case of unilateral dysplastic hip involvement [11,12,13]. However, the restoration of the anatomic hip COR at the true acetabulum can be challenging because of soft tissue retraction, an increased risk of neurological compromise after surgery, and above all for the acetabular supero-lateral bone defect that can compromise implant stability and integration [9,14]. Bone defect in the Crowe III hip can be managed by different solutions that allow COR restoration by the use of dedicated implants and surgical techniques, including bone autograft and allograft, 3D customized hip implants, and metal augments [15,16,17,18]. All these solutions restore the anatomical center of rotation of the hip, even though metal augments and 3D custom implants do not preserve bone stock and may complicate a subsequent revision surgery [19]. On the other hand, bone grafts allow the management of the bone defect by preserving or increasing the bone stock at the acetabular level. However, while autograft is supposed to osteointegrate and be available for structural support in future revision surgery, several authors criticize the use of allograft bone for the risk of graft resorption overtime with implant mobilization and failure [20].

In this scenario, femoral head autograft bone used to repair the acetabular defect could be an effective choice to allow an adequate coverage of the cup. It might promote bone stock restoration for an eventual revision surgery; however, there is a debate on the risk of graft resorption even for femoral autograft in hips affected by DDH [21]. The aim of this study is to evaluate the effectiveness of the femoral head autograft technique to restore the superolateral bone defect, and to determine its effectiveness in lowering the COR of the THA joint in Crowe III DDH patients treated by THA performed through the Direct Anterior Approach (DAA).

## 2. Materials and Methods

Between 2016 and 2022, 8 patients (9 implants) with Crowe III dysplastic hips and osteoarthritis due to DDH were operated on THA by DAA at the authors’ institution. 7 patients had unilateral joint involvement and in one it was bilateral. The study population represented 39.1% (9 of 23) of all the Crowe III patients operated in the same timeframe. Patient selection included maximum leg length discrepancy of 3.5 cm in patients with unilateral involvement, and they were all treated surgically only at the level of the hip, without performance of a femoral shortening osteotomy. Clinically, they presented severe hip pain and functional compromise.

All the study patients were treated by the same implants, whose ancillary instrumentation is dedicated to the DAA: for the cup a hemispheric 3D printed cementless porous titanium shell, Mpact 3D, was used; for the stem the AMIStem (MEDACTA International-Swiss) a double tapered cementless, hydroxyapatite covered, classified as type 3c according to Mont’s et al. [22], was implanted. Liner and femoral head were in 4th generation ceramic.

All the patients were evaluated clinically in terms of pre- and post-operative HHS, and in terms of surgical related parameters (blood loss, surgical time) and surgery-related complications. Radiological parameters that were evaluated included cup inclination, stem alignment, center-edge (CE) angle, limb length discrepancy before and after surgery, and coverage.

### 2.1. Surgical Technique

The patient lies supine on a specific traction table with perineal support, that allows hip flexion, extension, abduction and adduction, and rotation. A standard DAA is performed to reach the pathological joint; the true acetabulum is detected and freed from the soft tissues; full exposure of the deformed acetabulum in high dislocated Crowe III DDH hips requires the release of the proximal portion of tensor fascia latae to expose the iliac wing. After neck osteotomy, the femoral head is removed and stored to be used as autograft.

**Figure 1:**
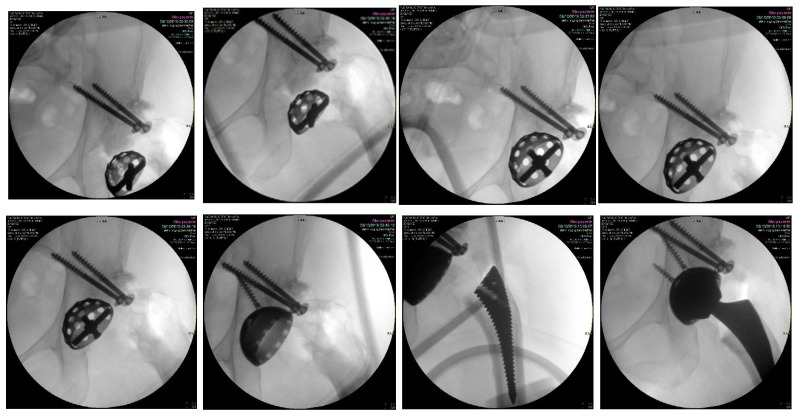
Progressive reaming in the true acetabulum, with the autograft kept in place by two screws, to improve the superior horizontal coverage.

Once fully exposed, it is possible to appreciate the deformation of the acetabular cavity, which misses its superolateral portion; usually, in Crowe III hips, the superior posterior rim of the acetabulum is eroded with severe bone defect and low coverage for cup implant. A small reamer is used to check the true acetabulum by fluoroscopy, whose position is referred to the tear drop height. To promote coverage at the pathological acetabulum, a femoral head autograft is prepared exposing the cancellous bone on one side by cortical bone removal to promote graft incorporation. The aspect of the autograft with cancellous bone is placed facing the acetabular cavity to ease reaming, while the portion of the graft with cortical bone is left external to give stability by the use of screws. Bone graft is modeled and posed in place at the posterior-superior rim were the defect lies, and it is secured by three 2 mm K-wires. Under fluoroscopic control, 2 cannulated partially-threated screws are used to secure the graft to the iliac bone (Figure 1). 

**Figure 2:**
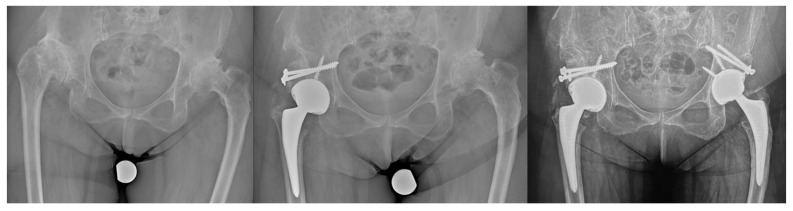
Dysplastic hip patient with bilateral sub dislocation, classified as Crowe III, treated by two stage surgery, restoring the anatomic hip center and repairing the supero-lateral bone defect with femoral head autograft stabilized by 2 screws.

Progressive reaming is performed with the autograft in place checking for the correct deepening and orientation of the reamers. A 3D porous coated titanium cup is impacted, and the primary stability of the implant is improved by 2 cancellous screws (Figure 2).

### 2.2. Radiological Assessment

DDH was classified according to Crowe et al. [1] on antero-posterior hip X-rays; only Crowe III hips with femoral head subluxation of 75–100% from the true acetabulum were selected and included in the study. LLD was measured pre and postoperatively, and it was calculated as the distance between the apex of lesser trochanter and the intersection of the line passing through the tear-drops, calculating the difference between the two legs.

**Figure 3:**
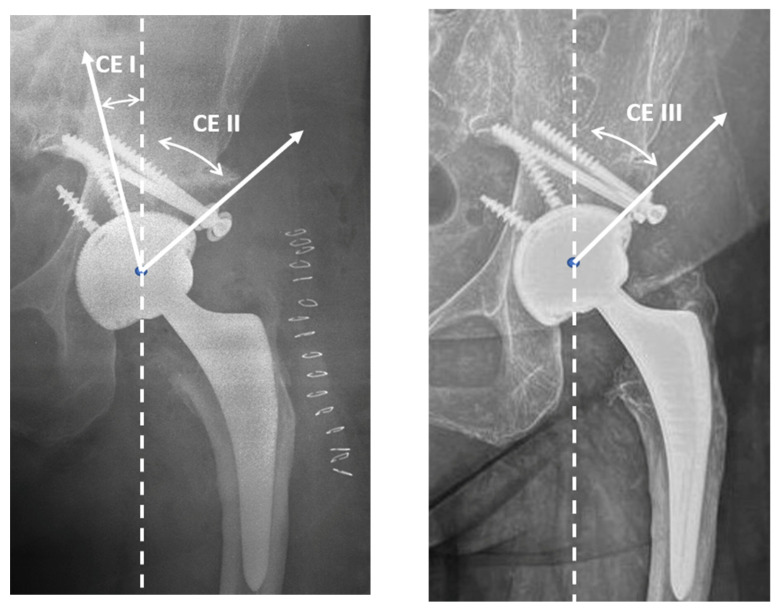
Center-edge angle: CE I, CE II, CE III.

Methods for determining center-edge (CE) angles (I to III) is shown in Figure 3, outlining the radiograph depicting method of evaluating graft coverage as described by Kim and Kadowaki [3]: (a) CE-I: the angle between a vertical line through the center of the femoral head and the lateral edge of the native acetabulum, which was also the medial edge of the graft, immediately postoperatively; CE-II: the angle between a vertical line through the center of the femoral head and the lateral edge of the graft bone immediately postoperative; (b) CE-III: the angle between a vertical line through the center of the femoral head and the lateral edge of the graft at the final postoperative visit [23].

**Figure 4:**
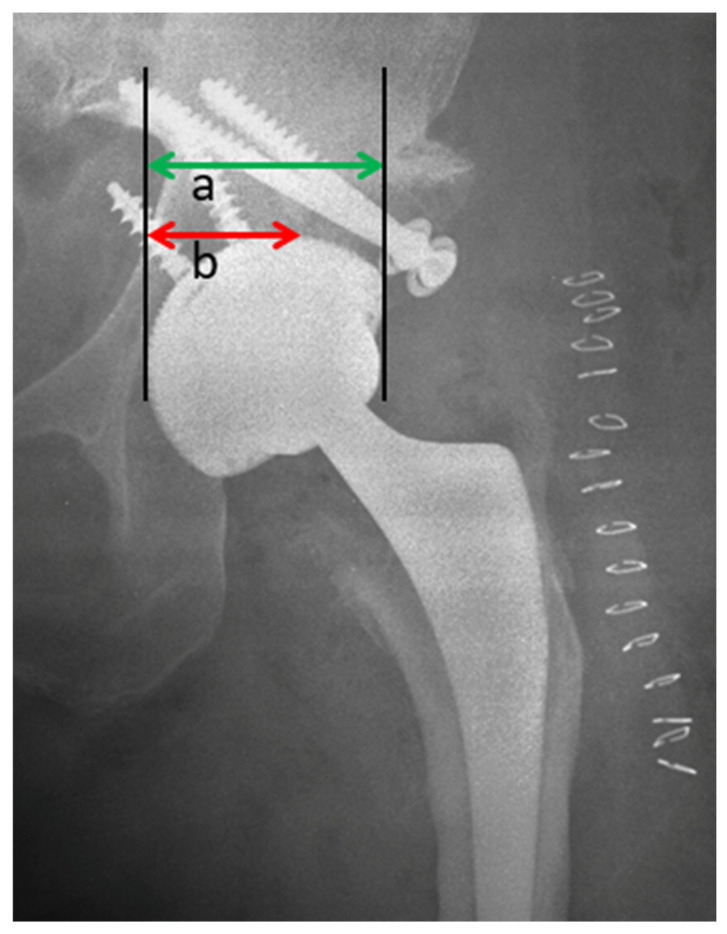
Horizontal Coverage defined as the: (horizontal host bone distance (**b**)/horizontal distance between the medial and lateral borders of the cup (**a**)) × 100.

Method for determining the horizontal coverage is shown in Figure 4; the radiograph depicts the method for measuring the percentage of horizontal coverage over the cup, calculated as: (horizontal host bone distance (b)/horizontal distance between the medial and lateral borders of the cup (a)) × 100. At the final follow-up, the horizontal host bone distance (b) included both graft and host bone, if union occurred [5].

Cup inclination was measured on anterior-posterior X-ray as the angle between the line tangent to the border of the acetabular cup and a line parallel to the horizontal plane, and it was compared to Lewinnek’s safe zone, which is 40 ± 10°. Stem alignment is the angle between the axis of the stem and of the femur; it was considered good when the angle between the axis of the stem and that of the femur was 0 ± 5°; above or below the range, the implant alignment was considered in varus or valgus, respectively. In patients who performed an evolutive CT to follow-up the autograft integration, cup anteversion was measured with respect to the sagittal plane measured on CT transverse images.

### 2.3. Clinical Assessment

For each patient, preoperative and postoperative visual analog pain score (VAS) and Harris Hip Score (HHS) were recorded. Preoperative ASA—American Society of Anesthesiologists—Score was collected. During in hospital stay, blood transfusions counted in blood units were collected for each patient. Surgical time was recorded. Both intraoperative and postoperative complications were considered, as well as causes of failure and related surgical revision management.

### 2.4. Statistical Analysis

The analyses were conducted comparing the groups with t-student test; significance was set at *p*-value < 0.05. (SPSS 14.0, version 14.0.1 (SPSS Inc., Chicago, IL, USA).

## 3. Results

A total of nine hips performed in eight patients, all classified by Crowe Classification as III types, were selected for the study, with an average follow-up of 4.8 years (range, 2–6 years). The population had an average age of 57.7 years (range, 49–72 years), a mean BMI of 25.7 kg/m^2^ (range, 22.4–31.2), with a median ASA score of 2 (range, 1–3). Two patients in their anamnesis reported surgical treatment in childhood, with only the open reduction of proximal femur dislocation; no osteotomy was performed in these patients. Three complications were recorded in our study population, two of which occurred in the same patient: one patient reported an intraoperative peri-prosthetic fracture managed by two metal wirings; the other one had implant dislocation occurring three months after surgery, which was managed by close reduction that caused a peri-prosthetic fracture, managed by a primary implant stem revision and metal wiring.

### 3.1. Clinical Results

Clinical data showed an improvement from pre-operative and post-operative outcomes in VAS, from 6.7 ± 2.3 to 1.7 ± 1.2 (*p* < 0.05), and in HHS (from 41.3 ± 9.4 to 92.7 ± 7.2 at least follow-up). Surgical time averaged 115.6 ± 21.2 min. Median blood transfusion in the perioperative period for each patient was 1 ± 1 Blood Units (Table 1).

Implant sizes included (Table 2) three 46 mm, four 48 mm, one 50 mm, and one 52 mm cups, with ceramic liner and femoral head sized 28 mm (*n* = 3), 32 mm (*n* = 5; 4 in 48 mm and 1 in 50 mm cups), and one 36 mm head for a 52 mm cup. Concerning the stem, in no patients the femoral pathological anatomy required the use of a conical stem to achieve the desired stability and version. Two stems measured size 1, four stems were size 2, and two were size 3.

### 3.2. Radiographic Results

Cups were placed in the tolerance range (Lewinnek’s safe zone 40 ± 10°) in all patients, with an average of 39.5 ± 7.5°. Stem alignment was in the tolerance range (±5° varus/valgus) for all the implants, with an average of 1.5 ± 2.3° valgus. LLD showed an overall average preoperative value of −29.5 ± 10.5 mm, with a significant improvement to −2.5 ± 6.4 mm (*p* = 0.023). In six patients who performed a post-operative CT scan, cup anteversion was measured with an average angle of 17.2 ± 5.1°.

The mean initial coverage evaluated as the percentage of the horizontal bone host was 52.1 ± 7.1%, while the mean final coverage in the last post-operative X-ray from femoral graft bone was 97.0 ± 2.5% with an average improving percentage of 44.5%. The average CE improved from −9.5 ± 5.2° (CE I) to the immediate post-operative (CE II) of 40.6 ± 8.2°. At the final follow up of average 24 months, CE III showed an average 38.6 ± 6.2°, with an average loss of 2.0°.

## 4. Discussion

The most important finding of the current study is that acetabular bone defect in Crowe III DDH can be efficiently managed by massive autograft femoral head during THA performance by DAA. The massive bone grafting allowed an adequate and long-lasting coverage of the cup with COR positioning at the native acetabulum.

High dysplastic hips with osteoarthritis can be managed by THA through different approaches. Most available literature reports good outcomes, in terms of clinical and radiological improvement, when THA is performed by direct lateral or postero-lateral approach [24,25,26,27]. Only recently, studies investigating the performance of THAs in Crowe III and IV hips by DAA have been reported. This is secondary to the spreading use of DAA in minimally invasive THA surgery; despite its diffusion in primary hip replacements, DAA allows an excellent exposure of all the elements of the pathological anatomy of DDH, mainly soft tissues including capsule, abductors and iliopsoas muscles, and proximal femur and true acetabulum. Most importantly, DAA gives full exposure to the acetabular ring for the optimal assessment of the bone defects, improving the chances of an effective management [28,29,30,31].

A lot of technological efforts have been applied to the management of acetabular bone defects, including the development of modular cups with augments, the use of buttress or jumbo cups, and 3D printed custom cups. However, the issue of revision of such implants in case of failure is still under debate [32,33,34]. A clear alternative to restore the bone stock at the acetabular level is the use of bone graft, either autologous or allogeneic, to restore the integrity of the acetabular ring [15,16] with the aim to provide full coverage to the cup while restoring a more physiological COR of the prosthetic hip.

The most straightforward technique, when possible, uses the patient’s femoral head to fill the superolateral bone defect to promote osteointegration and limit bone reabsorption [31]. Only a few authors up to date reported the results of the femoral head autograft technique performed by DAA [35,36]. Taylor et al. [31], in their study on dysplastic hips, reported of 41 patients operated on by this technique through DAA approach. Of these, 18 were Crowe III hips. At average 3.8 year follow up they found a good recovery of LLD by true acetabular cup placement while providing an excellent bone coverage to the implant, with an average horizontal coverage corrected from a preoperative value of 54% to a postoperative 98%. Moreover, minimal bone resorption was observed overtime, showing no significant differences among CE II and CE III at the last available follow-up. Viamont-Guerra et al. [28], in their study on 20 Crowe III patients, showed a minimal (2.8%) average reduction of CE III compared to CE II in a mean follow up of 8.4 years, confirming that bone autograft can properly integrate promoting implant coverage. Oinuma et al. [36] reported the results of bone graft to fill superior-lateral acetabular defect, together with subtrochanteric shortening osteotomy to perform THAs in 12 hips of nine patients with completely dislocated Crowe IV hips. Radiographic follow up of 3.7 years showed neither a radiolucent line nor loosening in this series. Our data support the use of autologous bone graft coverage of the acetabular defect in Crowe III patients. We achieved a horizontal implant bone coverage of 52.1 ± 7.1%, and an average final coverage at the last post-operative X-ray with a mean follow up of 24 months of 97.0 ± 2.5% with a long-lasting average improvement of 44.5%; at the same time, bone reabsorption calculated from CE III averaged only 2° at the last follow-up.

Cup placement is an important radiological parameter in determining the safest position to reduce the risk of dislocation of THAs [37]. As Lewinnek proposed in his studies, a desirable placement should be with a coronal inclination of 40° with respect to the plane passing by the radiological teardrops. Correct cup placement in the tolerance range of the Lewinnek safe zone, performed in patients with Crowe III hips, is associated with a low rate of dislocations [38,39]. Inclination alone is not sufficient to decrease the risk of dislocation, and it is important to associate proper cup anteversion. In literature, there is not a clear safe zone for cup anteversion, but most authors agree with a placement between 5–30° [40]. In the current study, cups coverage and anteversion fall within the tolerance range in all the patients, and only one dislocation was observed in a patient with an implant that had a cup inclination of 43° and anteversion of 18.1°.

LLD is a major issue in the high dysplastic Crowe III hips, because subluxation with upward and lateral femoral head migration generates a clinical and radiological LLD [41], causing patients’ dissatisfaction and functional impairment because of abductor muscles disfunction: LLD improvement with the restoration of eumetry improves functional and clinical outcomes [42]. Some authors reported high hip center THA placement to be a viable option in Crowe III and IV hips, reporting acceptable functional outcomes in terms of pain; however, this surgery is associated to a persistency of LLD > 10 mm in most patients [43,44]. Cup placement at the true acetabulum reduces LLD even in high dysplastic hips, giving the implant more favorable biomechanics [42]. Taylor et al. [31], in a study on 18 patients operated on using THA for Crowe III hips, reported the restoration of the physiological COR by cup placement at the true acetabulum, reducing LLD from an average 21.8 mm to 1.6 mm at the final follow-up of 3.8 years. Viamont-Guerra et al. [28], in their study of Crowe III and IV patients performing THA, obtained LLD correction with an average LLD of 2.5 mm at final follow-up. Our study reports similar findings, with a starting pre-operative LLD of 29.1 mm, reduced after THA to an average 1.5 mm.

High dysplastic hips, with severe osteoarthritis and unfavorable abductor muscle lever, are typically associated to an important functional limitation in walking, with lameness and reduced quality life [35]. Hamrayev et al., in his study on 22 patients with Crowe III hips operated on with THA by DAA, obtained an important improvement in post-operative functional scores, including HHS, when compared to the pre-operative functional status. Other authors [31,45] reported favorable outcomes of THAs performed in Crowe III and IV hips by DAA. Clinical results showed a marked increase in HHS, with good patient satisfaction. According to our findings, HHS showed an improvement from 41.3 ± 9.4 to 92.7 ± 7.2 at the last follow-up, supporting recent literature from DAA studies.

Limitations of our study include its retrospective nature, the absence of a comparison group, and the small study cohort. However, given the relative rarity of the disease and the selective indications to the surgical technique, and seeing the agreement with current literature published by surgeons with high volume THAs performed by DAA, our findings support the use of femoral bone autograft to promote cup stability, coverage, and osteointegration in Crowe III hips requiring THA.

## 5. Conclusions

In conclusions, we found that THA performed by DAA, with bone autograft reconstruction by an appropriately molded femoral head secured by screws, is effective in improving implant cup coverage by filling the bone defect characteristic of Crowe III hips. This result is kept overtime, even at middle-term follow up. The restoration of a physiological COR provided satisfactory results in terms of return to function, with LLD and lameness correction.

## Figures and Tables

**Table 1:** Summary demographic and clinical parameters. BMI: Body Mass Index; VAS: Visual analog pain score; ASA: American Society of Anesthesiologists Score.

	Age	BMI	ASA	VAS PRE	VAS POST	Surgical Time	Blood Transfusions
Pt 1	49	22.4	1	9	0	131	1
Pt 2	60	31.2	3	7	1	98	0
Pt 3	54	23.7	2	5	2	101	2
Pt 4	63	27.6	2	6	1	111	1
Pt 5	65	28.1	2	7	3	121	1
Pt 6	72	24.8	1	8	1	126	0
Pt 7	51	27.9	1	6	1	99	1
Pt 8—1°	52	23.1	2	6	2	124	1
Pt 8—2°	54	22.9	3	7	0	130	2
Total	57.7 y	25.7	1.9	6.7	1.2	115.6	1

**Table 2:** Summary of implant specifications.

Cup Size	Femoral Head Diameter	Femural Stem Size
46 mm (*n* = 3)	28 mm	1 (*n* = 2)
48 mm (*n* = 4)	32 mm	2 (*n* = 4)
50 mm (*n* = 1)	32 mm	3 (*n* = 3)
52 mm (*n* = 1)	36 mm	

## Data Availability

All collected data are reported in the current manuscript.

## References

[1] Crowe J.F., Mani V.J., Ranawat C.S. (1979). Total hip replacement in congenital dislocation and dysplasia of the hip. J. Bone Jt. Surg..

[2] Zhu J., Fernando N.D. (2020). Classifications in Brief: The Hartofilakidis Classification of Developmental Dysplasia of the Hip. Clin. Orthop. Relat. Res..

[3] Yang S. (2012). Total hip arthroplasty in developmental dysplasia of the hip: Review of anatomy, techniques and outcomes. World J. Orthop..

[4] Demirel M., Kendirci A.S., Saglam Y., Ergin O.N., Sen C., Öztürk I. (2022). Comparison of High Hip Center versus Anatomical Reconstruction Technique in Crowe Types II and III Developmental Dysplasia of the Hip: A Retrospective Clinical Study. Acta Chir. Orthop. Traumatol. Cech..

[5] Stirling P.B., Viamont-Guerra M.-R., Strom L.B., Chen A.F.M., Saffarini M.M., Nover L.M., Laude F. (2021). Does Cup Position at the High Hip Center or Anatomic Hip Center in THA for Developmental Dysplasia of the Hip Result in Better Harris Hip Scores and Revision Incidence? A Systematic Review. Clin. Orthop. Relat. Res..

[6] Di Martino A., Coppola M.A.R., Bordini B., Stefanini N., Geraci G., Pilla F., Traina F., Faldini C. (2021). Clinical and radiological outcomes of total hip arthroplasty in patients affected by Paget’s disease: A combined registry and single-institution retrospective observational study. J. Orthop. Traumatol..

[7] Ito H., Matsuno T., Minami A., Aoki Y. (2003). Intermediate-term results after hybrid total hip arthroplasty for the treatment of dysplastic hips. J. Bone Jt. Surg..

[8] Schutzer S.F., Harris W.H. (1994). High placement of porous-coated acetabular components in complex total hip arthroplasty. J. Arthroplast..

[9] Yang T.-C., Chen C.-F., Tsai S.-W., Chen W.-M., Chang M.-C. (2017). Does restoration of hip center with subtrochanteric osteotomy provide preferable outcome for Crowe type III–IV irreducible development dysplasia of the hip?. J. Chin. Med. Assoc..

[10] Doehring T., Rubash H., Shelley F., Schwendeman L., Donaldson T., Navalgund Y. (1996). Effect of superior and superolateral relocations of the hip center on hip joint forces: An experimental and analytical analysis. J. Arthroplast..

[11] Bicanic G., Delimar D., Delimar M., Pecina M. (2009). Influence of the acetabular cup position on hip load during arthroplasty in hip dysplasia. Int. Orthop..

[12] Marangoz S., Atilla B., Gök H., Yavuzer G., Ergin S., Tokgözoğlu A.M., Alpaslan M. (2010). Gait Analysis in Adults with Severe Hip Dysplasia before and after Total Hip Arthroplasty. HIP Int..

[13] Nie Y., Ning N., Pei F., Shen B., Zhou Z., Li Z. (2017). Gait Kinematic Deviations in Patients with Developmental Dysplasia of the Hip Treated with Total Hip Arthroplasty. Orthopedics.

[14] Wen X., Zuo J., Liu T., Gao Z., Xiao J. (2021). Bone defect map of the true acetabulum in hip dysplasia (Crowe type II and III) based on three-dimensional image reconstruction analysis. Sci. Rep..

[15] Loganathan B., Sharma V., Kumar M.R., Soundarapandian S., Marothi DP S., Sharma K. (2020). Acetabulum Reconstruction with TantalumCup and Augments in Dysplastic Hip Type 4 using 3D Printing Technology. J. Orthop. Case Rep..

[16] Yin S., Zhong H., Li R., Mou P., Yang J. (2018). Effectiveness of autologous femoral head bone graft in total hip arthroplasty for Crowe type III developmental dysplasia of hip with acetabular bone defect. Zhongguo Xiu Fu Chong Jian Wai Ke Za Zhi.

[17] Papalia R., Di Martino A., Caldaria A., Zampogna B., Denaro V. (2019). Outcomes of neck modularity in total hip arthroplasty: An Italian perspective. Musculoskelet. Surg..

[18] Di Martino A., Castagnini F., Stefanini N., Bordini B., Geraci G., Pilla F., Traina F., Faldini C. (2021). Survival rates and reasons for revision of different stem designs in total hip arthroplasty for developmental dysplasia: A regional registry study. J. Orthop. Traumatol..

[19] Anderl C., Steinmair M., Hochreiter J. (2022). Bone Preservation in Total Hip Arthroplasty. J. Arthroplast..

[20] Shinar A.A., Harris W.H. (1997). Bulk Structural Autogenous Grafts and Allografts for Reconstruction of the Acetabulum in Total Hip Arthroplasty. Sixteen-Year-Average Follow-up*. J. Bone Jt. Surg..

[21] Abdullah K.M., Hussain N., Parsons S.J., Porteous M.J., Atrey A. (2018). 11-Year Mean Follow-Up of Acetabular Impaction Grafting with a Mixture of Bone Graft and Hydroxyapatite Porous Synthetic Bone Substitute. J. Arthroplast..

[22] Khanuja H.S., Vakil J.J., Goddard M.S., Mont M.A. (2011). Cementless Femoral Fixation in Total Hip Arthroplasty. J. Bone Jt. Surg..

[23] Kim M., Kadowaki T. (2010). High Long-term Survival of Bulk Femoral Head Autograft for Acetabular Reconstruction in Cementless THA for Developmental Hip Dysplasia. Clin. Orthop. Relat. Res..

[24] Sofu H., Kockara N., Gursu S., Issin A., Oner A., Sahin V. (2015). Transverse Subtrochanteric Shortening Osteotomy During Cementless Total Hip Arthroplasty in Crowe Type-III or IV Developmental Dysplasia. J. Arthroplast..

[25] Şen C., Bilsel K., Elmadağ M., Güneş T., Saygi B. (2016). Acetabuloplasty at the Anatomic Centre for Treating Crowe Class III and IV Developmental HIP Dysplasia: A Case Series. HIP Int..

[26] Benazzo F.M., Piovani L., Combi A., Perticarini L. (2015). MODULUS Stem for Developmental Hip Dysplasia: Long-term Follow-up. J. Arthroplast..

[27] Risitano S., Piccato A., Fusini F., Rissolio L., Marcarelli M., Bosa G., Indelli P.F. (2022). Direct anterior approach in total hip arthroplasty: Influence of stem length on clinical and radiological outcomes at medium-term follow-up. Musculoskelet. Surg..

[28] Viamont-Guerra M.-R., Chen A.F., Stirling P., Nover L., Guimarães R.P., Laude F. (2020). The Direct Anterior Approach for Total Hip Arthroplasty for Severe Dysplasia (Crowe III and IV) Provides Satisfactory Medium to Long-Term Outcomes. J. Arthroplast..

[29] Liu Z., Bell C.D., Ong A.C., Wu S., Li Z., Zhang Y. (2020). Direct anterior approach total hip arthroplasty for Crowe III and IV dysplasia. Arthroplast. Today.

[30] Vanlommel J., Sutter M., Leunig M. (2020). Total hip arthroplasty using the direct anterior approach and intraoperative neurophysiological monitoring for Crowe III hip dysplasia: Surgical technique and case series. Acta Orthop. Belg..

[31] Taylor A.J., Runner R.P., Kay R.D., Najibi S. (2022). Femoral Head Autograft Can Reliably Reconstruct Dysplastic Acetabula Through the Direct Anterior Approach for Total Hip Arthroplasty. Arthroplast. Today.

[32] Lee J.-M., Kim T.-H. (2018). Acetabular Cup Revision Arthroplasty Using Morselized Impaction Allograft. Hip Pelvis.

[33] García-Cimbrelo E., García-Rey E. (2014). Bone Defect Determines Acetabular Revision Surgery. HIP Int..

[34] Chiarlone F., Zanirato A., Cavagnaro L., Alessio-Mazzola M., Felli L., Burastero G. (2020). Acetabular custom-made implants for severe acetabular bone defect in revision total hip arthroplasty: A systematic review of the literature. Arch. Orthop. Trauma Surg..

[35] Yildirim T., Guclu B., Karaguven D., Kaya A., Akan B., Cetin I. (2015). Cementless Total Hip Arthroplasty in Developmental Dysplasia of the Hip with End Stage Osteoarthritis: 2–7 Years’ Clinical Results. HIP Int..

[36] Oinuma K., Tamaki T., Miura Y., Kaneyama R., Shiratsuchi H. (2014). Total Hip Arthroplasty with Subtrochanteric Shortening Osteotomy for Crowe Grade 4 Dysplasia Using the Direct Anterior Approach. J. Arthroplast..

[37] Lewinnek G.E., Lewis J.L., Tarr R.I.C.H.A.R.D., Compere C.L., Zimmerman J.R. (1978). Dislocations after total hip-replacement arthroplasties. J. Bone Joint Surg. Am..

[38] Hamrayev A.J., Buyukkuscu M.O., Misir A., Gursu S.S. (2020). The fate of femoral head autograft in acetabular reconstruction in dysplastic hips at midterm. J. Orthop. Surg..

[39] Goto E., Umeda H., Otsubo M., Teranishi T. (2021). Cemented acetabular component with femoral neck autograft for acetabular reconstruction in Crowe type III dislocated hips. Bone Jt. J..

[40] Seagrave K.G., Troelsen A., Malchau H., Husted H., Gromov K. (2017). Acetabular cup position and risk of dislocation in primary total hip arthroplasty. Acta Orthop..

[41] Portinaro N., Case R., Gargan M. (1999). Pathological Anatomy of Developmental Dysplasia of the Hip Joint. HIP Int..

[42] Abolghasemian M., Samiezadeh S., Jafari D., Bougherara H., Gross A.E., Ghazavi M.T. (2013). Displacement of the Hip Center of Rotation After Arthroplasty of Crowe III and IV Dysplasia: A Radiological and Biomechanical Study. J. Arthroplast..

[43] Shen J., Sun J., Du Y., Zhang B., Li T., Zhou Y. (2021). Functional and radiographical results of asymmetrically reconstructed total hip arthroplasty in patients with bilateral dysplastic arthritic hips with one hip Crowe II–III and the other Crowe IV: A retrospective cohort study. J. Orthop. Traumatol..

[44] Shen J., Sun J., Ma H., Du Y., Li T., Zhou Y. (2020). High Hip Center Technique in Total Hip Arthroplasty for Crowe TypeII–III Developmental Dysplasia: Results of Midterm Follow-up. Orthop. Surg..

[45] Song J.H., Ahn T.S., Yoon P.W., Chang J.S. (2017). Reliability of the acetabular reconstruction technique using autogenous bone graft from resected femoral head in hip dysplasia: Influence of the change of hip joint center on clinical outcome. J. Orthop..

